# An Influenza Virus Hemagglutinin-Based Vaccine Platform Enables the Generation of Epitope Specific Human Cytomegalovirus Antibodies

**DOI:** 10.3390/vaccines7020051

**Published:** 2019-06-14

**Authors:** Mohammad Amin Behzadi, Kathryn R. Stein, Maria Carolina Bermúdez-González, Viviana Simon, Raffael Nachbagauer, Domenico Tortorella

**Affiliations:** 1 Department of Microbiology, Icahn School of Medicine at Mount Sinai, New York, NY 10029, USA; ma.behzadi@mssm.edu (M.A.B.); Kathryn.Stein@icahn.mssm.edu (K.R.S.); maria.bermudez-gonzalez@mssm.edu (M.C.B.-G.); viviana.simon@mssm.edu (V.S.); 2 The Global Health Emerging Pathogens Institute, Icahn School of Medicine at Mount Sinai, New York, NY 10029, USA; 3 Division of Infectious Disease, Department of Medicine, Icahn School of Medicine at Mount Sinai, New York, NY 10029, USA

**Keywords:** influenza virus, human cytomegalovirus, vaccine, humoral immunity, neutralization, hemagglutinin, gH envelope protein

## Abstract

Human cytomegalovirus (CMV) is a highly prevalent pathogen with ~60%–90% seropositivity in adults. CMV can contribute to organ rejection in transplant recipients and is a major cause of birth defects in newborns. Currently, there are no approved vaccines against CMV. The epitope of a CMV neutralizing monoclonal antibody against a conserved region of the envelope protein gH provided the basis for a new CMV vaccine design. We exploited the influenza A virus as a vaccine platform due to the highly immunogenic head domain of its hemagglutinin envelope protein. Influenza A variants were engineered by reverse genetics to express the epitope of an anti-CMV gH neutralizing antibody that recognizes native gH into the hemagglutinin antigenic Sa site. We determined that the recombinant influenza variants expressing 7, 10, or 13 residues of the anti-gH neutralizing antibody epitope were recognized and neutralized by the anti-gH antibody 10C10. Mice vaccinated with the influenza/CMV chimeric viruses induced CMV-specific antibodies that recognized the native gH protein and inhibited virus infection. In fact, the influenza variants expressing 7–13 gH residues neutralized a CMV infection at ~60% following two immunizations with variants expressing the 13 residue gH peptide produced the highest levels of neutralization. Collectively, our study demonstrates that a variant influenza virus inserted with a gH peptide can generate a humoral response that limits a CMV infection.

## 1. Introduction

Human cytomegalovirus (CMV) is a member of the β-herpesvirus family and is a widespread pathogen among the population [1]. CMV establishes latency and can periodically reactivate leading to enhanced morbidity and mortality in immunocompromised individuals such as newborns, organ transplant recipients, and AIDS patients. Congenital CMV infections are the leading cause of birth defects, affecting ~0.5%–1% of newborns with up to 40,000 new cases/year in the US [2,3]. Notably, 5%–10% of congenitally infected neonates have extensive CNS disorders in the form of encephalitis, deafness, upper motor neuron disorders, psychomotor retardation, myopathy, and choroidoretinitis [4]. These at-risk patient populations provide clear examples of an unmet medical need to identify safe and effective therapeutic strategies that limit CMV dissemination. CMV has been estimated to cost the US as much as $4.4 billion per year by a National Academy of Sciences report [5]. Although ganciclovir (GCV), valganciclovir, foscarnet, cidofovir, and most recently, letermovir [6,7] are approved as anti-virals, these therapeutics have limitations that preclude their long-term use including poor oral bioavailability, dose-related toxicity, and selection of drug resistant viral mutants [8]. There continues to be a major necessity for prevention and control of CMV infection and dissemination.

CMV virions contain a large (~236 kb) dsDNA genome. The virion envelope is studded with envelope proteins and complexes such as gB, gH/gL/gO, gH/gL/UL128/UL130/UL131a (pentamer, PC), and gN/gM that contribute to the wide cellular and tissue tropism of the virus [9]. In fact, entry into fibroblasts occurs at the cell surface by membrane fusion/macropinocytosis in a pH-independent manner involving gB and the gH/gL/gO complex. In contrast, entry into epithelial, endothelial, dendritic, and monocytic cells occurs within the endosome and/or by macropinocytosis in a pH-dependent manner facilitated by gB, the gH/gL/gO, and the PC [10]. Following the fusion event between the cellular and viral envelope, the capsid then traffics to the nucleus where the viral genome is deposited. The temporal expression of viral genes in an immediate early (IE), early, and late phase expression promotes virus replication and production of virions. The >185 viral proteins expressed during the CMV life-cycle are potential immune targets for both the humoral and cellular immunity. Immunoglobulins targeting envelope proteins may have the ability to limit virus infection and proliferation, while cellular immunity against endogenous and exogenous viral proteins would target virus-infected cells. Thus, an effective CMV vaccine may be required to induce both branches of the immune system to control virus infection and dissemination.

A CMV vaccine is a major public health priority to prevent diseases associated with congenital CMV infections and in hematopoietic stem cell and solid organ transplant recipients [11]. Vaccination of pregnant women would require a high safety profile to ensure that an immune response to the vaccine does not cause adverse reactions to the placenta or the fetus. Ideally, administration of a vaccine would occur prior to pregnancy to limit virus infection and dissemination to the fetus. Previous and current vaccine approaches have mostly focused on generating a humoral response against the gB envelope protein due to its involvement with virus entry and a T cell-mediated immune response against the immediate early gene IE1 and the tegument protein pp65 [12]. The diverse vaccine platforms include live-attenuated vaccines, DNA vaccines expressing CMV proteins, recombinant viral proteins, and vector-based vaccines [13]. Several vaccine trials using a recombinant gB protein with the MF59 adjuvant (gB/MF59) generated a humoral and cell-mediated immune response and showed some promise in reducing infection rates and duration of viremia, respectively [14,15,16]. Yet, the bivalent DNA vaccine ASP0113 that expresses gB and pp65 was ineffective at reaching the primary endpoint in a Phase II solid organ transplantation (SOT) and Phase III hematopoietic cell transplantation (HCT) clinical trials [12,17]. There are additional vaccine trials for HCT recipients that have focused on boosting T cell immunity including a chimeric peptide consisting of the pp65 fragment (PepVax) [18] and a vector-based vaccine in which the modified vaccinia Ankara (MVA) (Triplex) expresses viral genes pp65, IE1-exon4 and IE2-exons5 [19]. These studies report an expansion of CD8 T cells and for PepVax, reached its clinical outcome supporting previous findings that the adaptive arm of the immune system can control CMV proliferation [20]. A new approach for a CMV vaccine, referred to as a disabled infectious single-cycle (DISC) CMV variant regulates viral replication though a fusion protein consisting of an essential viral gene with the FKBP12 protein that is stable in the presence of the ligand Shield-1 [21,22,23]. In the absence of Shield-1, the chimeric genes are degraded and thus, halting virus replication. The CMV strain V160 consists of the AD169 strain with a repaired UL131a and FKBP12 fusion proteins of IE1/IE2 and the UL51 viral packaging protein and is currently in a Phase I clinical trial [11]. Collectively, these vaccine strategies are designed to enhance the immune response that occurs during a natural CMV infection.

Ideally, a CMV vaccine would generate an immune response against conserved viral epitopes that provides protection against primary and recurrent CMV infections. The CMV gH protein is an attractive target for such a vaccine because it is an essential component of the gH/gL/gO trimer and the gH/gL/UL128/UL130/UL1301a pentamer entry complexes providing virus tropism [24]. Neutralizing monoclonal antibodies (mAbs) directed to gH inhibited infection in various cell types including fibroblast and epithelial cells through interacting with both the trimer and pentamer complexes [25,26,27,28]. Importantly, recently identified neutralizing mAbs target a specific region of the CMV gH protein that is conserved in the majority of clinical CMV strains [25]. Thus, the anti-gH epitope sequence was cloned into a major Sa antigenic site of the influenza A virus hemagglutinin (HA) and rescued variant influenza viruses were used as vaccine antigens to generate a targeted humoral response against CMV gH. Influenza virus has been utilized as a vaccine vector for numerous pathogens including *Plasmodium* species and HIV [29]. In addition, recombinant influenza viruses that express a single-chain anti-CTLA4 antibody and GM-CSF show oncolytic potential [30,31]. The sera from animals immunized with the influenza/CMV variants recognized gH and inhibited virus infection in vitro in CMV infectivity assays. This is a proof-of-concept study for an influenza-based vaccination platform to generate a humoral response against a specific region of a viral envelope protein that limits a CMV infection.

## 2. Materials and Methods

### 2.1. Animal Study Approval

Animal experiments were performed in accordance with protocols approved by the Institutional Animal Care and Use Committee at the Icahn School of Medicine at Mount Sinai (IACUC-2015-0119).

### 2.2. Cell Lines, Antibodies, and Viruses

Human embryonic kidney 293T cells, Madin-Darby Canine Kidney (MDCK) cells, MRC5 lung fibroblasts and human U373-MG astrocytoma cells (utilized to stably express CMV gH [25]) were obtained from the American Type Culture Collection (ATCC, Manassas, VA, USA) and grown in Dulbecco’s modified Eagle’s medium (DMEM) supplemented with 10% fetal calf serum (HyClone, Logan, UT, USA) and 1% penicillin-streptomycin (GIBCO). Human retinal epithelial ARPE-19 cells (ATCC #CRL-2302) were cultured in DMEM and Ham’s F-12 (1:1). Influenza A/Puerto Rico/8/34 (H1N1, PR8) virus and rescued recombinant PR8 viruses were grown in 10-day-old specific pathogen-free (SPF) chicken eggs (Charles River Laboratories) were used in this study. The CMV neutralizing gH mAb 10C10 and gB mAb 2F4 was purified from hybridoma culture supernatant [25]. Both monoclonal antibodies recognize proteins in their native structure within the virion or virus infected cells and do not bind to denatured proteins as demonstrated by immunoblot analysis [25]. The mAbs CR9114, KB2, and PY102recognize the stalk (CR9114, KB2) or globular head (PY102) of the HA of PR8 (H1N1) influenza A virus [32]. CMV virus was propagated in MRC5 cells and virus from infected-cell supernatant and cell lysate (pooled) following sonication was purified by density gradient centrifugation by spinning at 20,000 r.p.m. at room temperature for 1.5 h over a 20% sorbitol cushion [33]. Infectious virus yield of AD169 (BADrUL131 C4) (a gift from Dr. Thomas Shenk, Princeton University) and TB40/E wt (a gift from Dr. Christian Sinzger, University of Medical Center Ulm) was assayed on MRC5 fibroblasts by median tissue culture infectious dose (TCID50).

### 2.3. Chimeric Influenza Virus Model

Amino acid sequence of PR8 HA (accession no.: AYA81842, PDB-3LZG) and CMV gH (accession no.: CAA00301, [25]) were acquired from the NCBI database. The protein sequences of CMV gH, PR8 HA, and the chimeric viruses HA/gH-7AA, HA/gH-10AA, and HA/gH-13AA were directly submitted to the SWISS-MODEL Automatic Modeling Model server (www.swissmodel.expasy.org) to search for the homologous contribution of the three-dimensional structure (3D) online. 3D structure models were generated using PyMOL (The PyMOL Molecular Graphics System, Version 2.0.1, Schrödinger, LLC, New Yor, NY, USA) for viewing the outputs produced by the SWISS-MODEL homology server.

### 2.4. Chimeric Influenza Virus Construction and Amplification

The recombinant PR8 chimera viruses HA/gH-7AA, HA/gH-10AA, and HA/gH-13AA were constructed using a reverse genetics system as previously described [34]. In brief, a previously described gH epitope of CMV (487-EIFIVET-493) recognized by mAb 10C10 was inserted into the Sa site of H1 molecule of influenza strain PR8. Constructed ambisense DNA plasmids were cloned into the cloning vector pDZ and transfected into 293T cells with a 7-segment 7 plasmid encoding the essential viral proteins and virus-like RNA of PR8. Scraped cells and supernatants were injected into 8–10 day old embryonated chicken eggs (Charles River Laboratories) for viral rescue at 37 °C for 48 h. Viruses were plaque purified on MDCK cells [35,36]. Individual plaques were picked and injected into embryonated eggs, and viral RNAs were extracted from the allantoic fluids and HA segments were Sanger sequenced. Viral titers were determined at specific time points using plaque assays on MDCK cells. Viruses were concentrated by ultracentrifugation through a 30% buffered sucrose cushion (Beckman L7-65 ultracentrifuge with SW-28 rotor at 25,000 rpm) and stored at −80 °C. The viruses were titered using 100 PFU as previously described [37].

### 2.5. Immunofluorescence Staining

HEK293T cells were grown in 24-well plates coated with poly-L-lysine (Sigma-Aldrich, St. Louis, MO, USA). Cells were transfected with each recombinant plasmid (500 ng) in optimized minimal essential medium (MEM) (Opti-MEM; Gibco) using Lipofectamine 2000 (Thermo Fisher Scientific, Waltham, MA, USA) at a 1:2 ratio, and incubated for 24 h at 37 °C. MDCK cells were also infected with rescued plaque purified viruses and incubated at 37 °C for 2 days. Following incubation, medium was removed and the cells were fixed with 4% paraformaldehyde in PBS for an hour at room temperature (RT). After the fixation process, the cells were washed with PBS, blocked with 4% BSA in PBS, and incubated with primary anti-HA stalk murine mAb KB2, or anti-gH CMV specific mAb 10C10, at a dilution of 1:500 for 2 h at 37 °C. Cells were washed five times with PBS and incubated at RT for 1 h with an anti-mouse Alexa Fluor-488 antibody (Life Technologies, Carlsbad, CA, USA) diluted 1:1000 in 1% BSA–PBS. Fluorescent signals were visualized and captured by using an Olympus 1X-70 microscope.

### 2.6. Influenza Virus Hemagglutination Inhibition Activity and Neutralization Assay

To determine the hemagglutination inhibition (HI) activity of CMV-specific mAb 10C10 against rescued chimeric viruses, an HI assay was performed as previously described [38,39,40]. Briefly, chicken red blood cells (Lampire) were washed in PBS and resuspended at a concentration of 0.05% hematocrit. 100 µg/mL of 10C10 was serially diluted 1:2 in 25 μL volumes across a 96-well V-bottom plate. Allantoic fluid containing PR8 wild-type or rescued chimera viruses were diluted to eight HA-units and then incubated in equal volumes to mAb (25 μL each) for 30 min at 25 °C. Chicken red blood cells were then added and HI titers were visually determined. Testing was performed in duplicate.

Neutralization assays are routine in lab and were performed as previously described [38,39,40]. Briefly, two-fold dilutions (starting from 100 µg/mL) of CMV neutralizing gH mAb 10C10, PY102 (PR8 anti-head specific mAb), CR9114 and KB2 (influenza HA anti-stalk specific mAbs) in sterile Opti-MEM (Invitrogen, Carlsbad, CA, USA) were mixed with 100 TCID50 of rescued influenza viruses. The mAbs-virus samples were then incubated at room temperature for 60 min to allow antibody binding. The mAbs-virus samples (100 µL) were then transferred to MDCK cells cultured in 96-well flat bottom plates. Following virus adsorption for 60 min, the mAbs-virus inocula were removed, and the MDCK cells were cultured for 2 days at 37 °C in Ultra-MDCK media (Lonza, Basel, Switzerland) supplemented with 1 µg/mL of TPCK-trypsin (Sigma-Aldrich, St. Louis, MO, USA). Virus production was determined by a hemagglutination assay that measures virus titer based on relative hemagglutination activity with chicken red blood cells. The neutralization titer was defined as the reciprocal of the highest dilution of mAb that neutralizes 100 TCID50 of influenza virus. Testing was performed in triplicate.

### 2.7. Animal Vaccinations

Twenty female BALB/c mice (6–8-week old, Jackson Laboratories) were divided into 4 groups (3 experimental and 1 control groups). Mice in the experimental groups were sequentially vaccinated with purified rescued mosaic viruses (10 µg, 5 × 10^7^ pfu) of admixed with Addavax (InvivoGen INC, San Diego, CA, USA), a squalene-based oil-in-water nano-emusion per the manufacturer’s instructions. The animals in the control group were vaccinated with PR-wt lacking any CMV peptide plus adjuvant. Each animal received four injections, 3 weeks apart, at a total volume of 50 µL (25 µL virus and 25 µL Addavax). The vaccination strategy utilizing a high antigen dose and adjuvant was chosen to elicit high levels of antibodies as a proof-of-principle. The virus was non-inactivated and could therefore undergo limited replication in the muscle tissue, potentially further boosting the immune response. About 200 µL of blood was collected from each animal from the submandibular vein prior to immunization. For immunization with whole CMV virus, mice were vaccinated with TB40/E wt (100 µg, 2 × 10^6^ pfu) followed by boost with 50 µg of virus preparation. All the injections were carried out intramuscularly using insulin syringes with a 29-gauge needle attached.

### 2.8. CMV Serum Inhibition Assay

CMV reporter strain AD169 (BADrUL131 C4) (MOI: 0.5) was incubated with serum (1:100, 1:500, and 1:1500/1:2500 dilutions), anti-gH (10C10) or anti-gB antibody (2 µg/mL) (1 h 4 °C), then added to ARPE-19 cells (1 × 10^4^ cell/well, 96-well plate). After incubation for 2 h (37 °C), inoculum was then replaced with new media. At 24 hpi, cells were fixed in 4% paraformaldehyde. Cells were stained with Hoechst 33342 (1 µg/mL, Molecular Probes) in PBS (15 min room temperature), before analysis by the Celigo Image Cytometer (Nexcellom Bioscience, Lawrence, MA, USA) for YFP intensity. All samples were tested in technical replicates of three. The inhibition assay is routine in the laboratory as previously characterized [25,33].

### 2.9. Flow Cytometry gH Binding Analysis

U373-expressing gH/L cells and control cells that do not express gH/gL were blocked with 1%BSA/PBS (20 min, 4 °C), incubated with serum (1:100 dilution), anti-gH (10C10) or anti-HA (PY102) antibodies (2 µg/mL) (1 h, 4 °C), and then incubated with Alexa Fluor-647 goat anti-mouse (1 µg/mL) (Thermo Fisher Scientific), before collection on an Intellicyt HTFC flow cytometer and quantification was determined using Flow Jo software (Tree Star, Inc, Ashland, OR, USA).

## 3. Results

### 3.1. Generation of Chimeric Influenza/CMV

**Figure 1:**
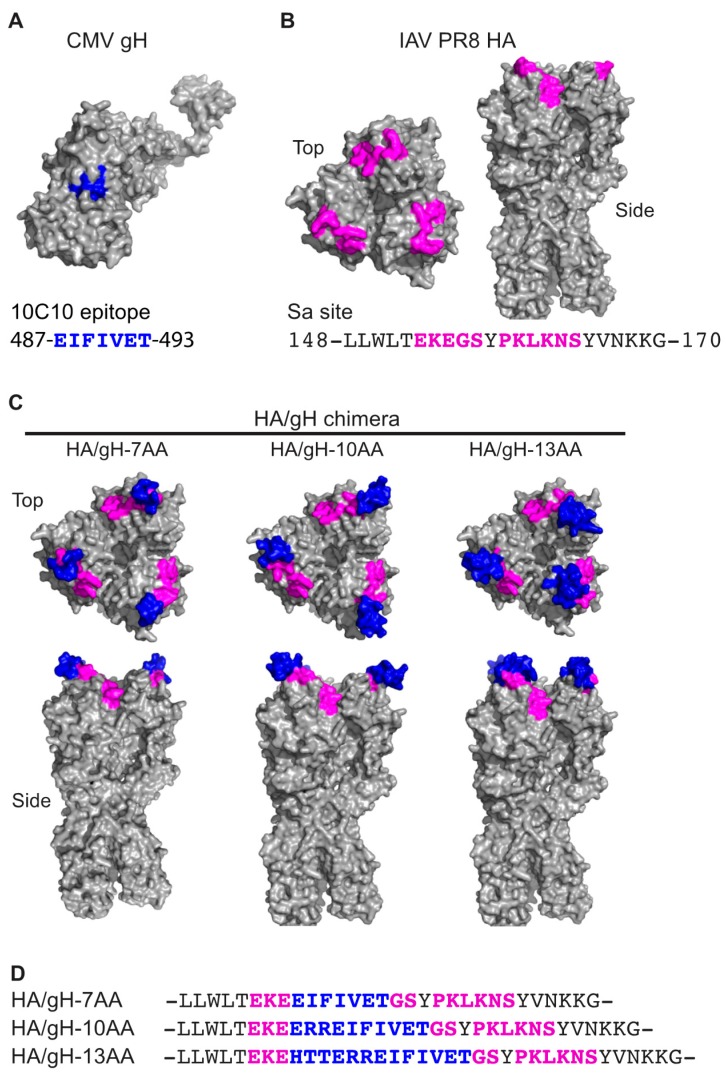
Model of the chimeric influenza hemagglutinin (HA)/CMV gH molecules. (**A**) Predicted structure of the CMV gH protein. Blue color indicates the mAb 10C10 recognition epitope. (**B**) Predicted structure of influenza A virus (IAV) PR8 HA trimer (PDB3LZG) (top view and side view) with the classically defined antigenic site Sa colored in pink. The AA numbering is based on Burke Reference Sequence Alignment for PR8 [43]. (**C**) Predicted structures of chimera HAs containing CMV gH epitope (7AA, 10AA, or 13AA, in blue) within Sa antigenic site (in pink). Modeling performed with PyMOL (The PyMOL Molecular Graphics System, Version 2.0.1, Schrödinger, LLC) [44]. Amino acids in gray are unchanged. (**D**) Amino acid sequences of the Sa antigenic site (in pink) of chimera IAVs (A/Puerto Rico/8/1934(H1N1)) with CMV gH epitopes (7AA, 10AA, or 13AA, in blue).

CMV expresses numerous envelope proteins that mediate virus binding and entry into diverse cell types [9]. Previous studies identified a monoclonal antibody (mAb) 10C10 that recognizes the native CMV gH protein and neutralizes virus infection by targeting a post binding step [25]. The mAb 10C10 epitope contains residues 481-HTTERREIFIVET-493 of gH, a region predicted to be within an alpha helix of Domain II of gH (Figure 1A). The 10C10 mAb recognizes gH within the virion and virus infected cells, but does not recognize gH as a denatured linear protein, suggesting that the mAb binds to a native epitope within gH [25]. In order to evaluate the potential of an influenza virus-based vaccine platform to generate a domain specific humoral response, a chimeric hemagglutinin (HA)/gH molecule was generated by introducing the 10C10 epitope of gH into the flexible region of the highly immunogenic antigenic Sa site of the A/Puerto/Rico/34 (PR8) influenza HA protein (Figure 1B) [41,42]. To enhance the probability of the proper folding of the chimeric HA/CMV protein, different gH peptides corresponding to 487-EIFIVET-493 (7 amino acids (7AA)), 484-ERREIFIVET-493 (10AA), and 481-HTTERREIFIVET-493 (13AA) were introduced between HA residues 155/156 [43]. The gH-7AA, -10AA, and -13AA peptides are predicted to be exposed on the surface of the HA molecule based on the HA native structure using Swiss-Model homology modeling (Figure 1C) [44].

**Figure 2:**
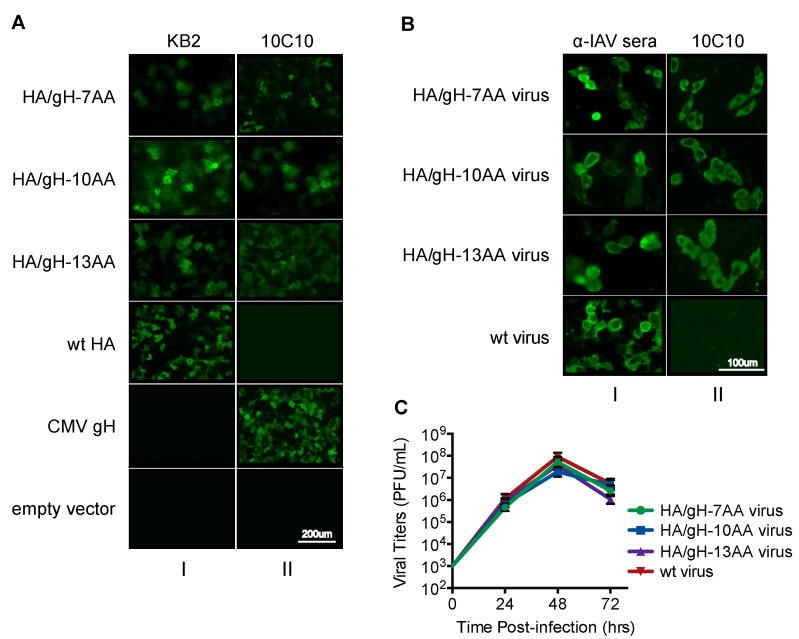
The gH peptide within the HA/gH chimeric viruses is conformationally similar to the native CMV gH protein. (**A**) Immunofluorescence staining of HEK293T cells transfected with plasmids encoding the HA/gH chimeras (HA/gH-7AA, HA/gH-10AA, and HA/gH-13AA), wild-type (wt) HA, and the full CMV gH protein were probed using anti-influenza A monoclonal antibody (KB2) and anti-CMV gH monoclonal antibody (10C10). Empty vector was utilized as a negative control. (**B**) MDCK cells infected with the chimeric HA/gH and wt-influenza viruses (MOI 0.25) were probed at 48 hpi with 10C10 and anti-influenza A polyclonal mouse serum (α-IAV sera). (**C**) Growth curves in MDCK cells of the chimera HA/gH viruses (HA/gH-7AA, HA/gH-10AA and HA/gH13AA) and of the wt-influenza virus (wt virus).

The generated chimeric viruses are referred to as HA/gH-7AA, HA/gH-10AA, and HA/gH-13AA (Figure 1D). We initially examined whether the gH peptide within the chimeric HA/gH molecule would be recognized by the anti-gH mAb 10C10 (Figure 2) [25]. HEK-293T cells transfected with plasmids encoding HA/gH-7AA, HA/gH-10AA, HA/gH-13AA, wild type (wt) HA, and the full length CMV gH protein were examined using immunofluorescence microscopy (Figure 2A). KB2, an HA stalk specific monoclonal antibody that recognizes a conformational epitope was utilized as a control to confirm proper folding of the HA/gH chimeric proteins. The KB2 antibody reacted to the HA/gH chimeras and the HA wt proteins validating the proper folding of the chimeric polypeptides (Figure 2A, column I). Importantly, the anti-gH 10C10 mAb reacted to the HA/gH chimeras and full-length gH molecule, while 10C10 did not bind to the HA wt protein (Figure 2A, column II). These findings indicate that the 10C10 epitope is exposed in the HA/gH chimera upon protein expression in cells.

Next, influenza viruses containing the HA/gH chimeras were rescued using a reverse genetics system [34]. Expression of the HA/gH protein encoded by chimeric viruses was evaluated in infected cells (Figure 2B). MDCK cells infected with HA/gH-7AA, HA/gH-10AA, HA/gH-13AA, and wt influenza virus were tested for HA/gH expression using immunofluorescence microscopy. Virus-infected cells were probed with polyclonal influenza antibodies (α-IAV sera) to confirm successful infection and the anti-gH 10C10 mAb. The α-IAV sera recognized virus-infected cells (Figure 2B, column I), while the 10C10 mAb only bound to the HA/gH molecules from cells infected with chimeric viruses (column II). The growth phenotype of the rescued viruses was assessed by growing the virus in embryonated chicken eggs for 24, 48, and 72 h, followed by titration in a plaque assay on MDCK cells (Figure 2C). The chimeric HA/gH-7AA, HA/gH-10AA, and HA/gH-13AA influenza viruses had similar growth kinetics as wt virus, implying that the introduction of the 7, 10, or 13 gH residues in the PR8 HA did not have a detrimental impact on viral proliferation. An insignificant difference among the titers of influenza virus (<1 log) was observed and does not reflect growth defects in variants. These results are consistent with previous influenza HA chimeric viruses where shorter peptide insertions (5 aa) within the HA did not affect viral growth [45]. These data demonstrate that the anti-gH epitope is exposed within the chimeric HA molecule during infection of the chimeric viruses.

### 3.2. A gH MAB Inhibits Hemagglutination and Neutralizes Chimeric Influenza Viruses

**Figure 3:**
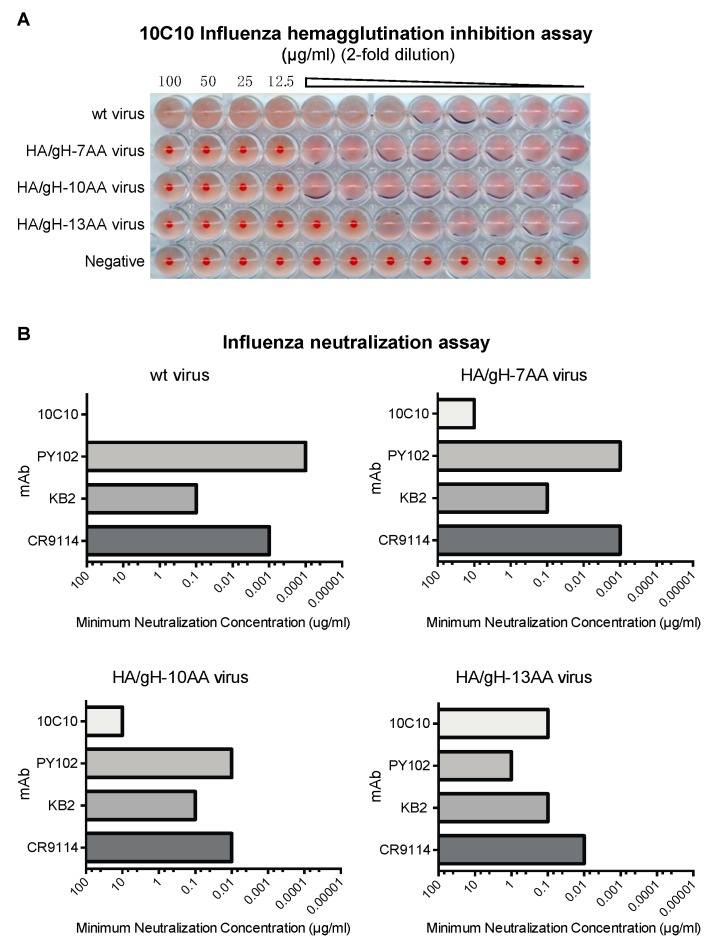
An anti-gH antibody neutralizes the chimeric influenza/CMV viruses. (**A**) Minimal hemagglutination inhibition assays was performed with increasing dilutions (two-fold dilutions starting from 100 µg/mL) of the anti-cytomegalovirus (CMV) gH monoclonal antibody (mAb) (10C10) against wild type influenza virus (wt virus) and chimera HA/gH influenza viruses (HA/gH-7AA, HA/gH-10AA and HA/gH-13AA). The accumulation of chicken red blood cells at the bottom of well indicates the lack of inhibition. (**B**) Wild type influenza virus (wt virus) and chimera HA/gH influenza viruses (HA/gH-7AA, HA/gH-10AA, and HA/gH-13AA) were subjected to influenza neutralization assay (in triplicate) with increasing concentrations (two-fold dilutions starting from 100 µg/mL) of mAb 10C10, PR8 head specific mAb (PY102), and HA stalk specific mAbs (KB2 and CR9114) against wt virus and chimera HA/gH viruses. The virus from infected cells was titered using a classical hemagglutination assay and plotted based on the minimal mAb concentration that neutralizes 100 TCID50 of influenza virus.

Anti-influenza antibodies directed against the Sa site interfere with binding of the HA to sialic acid, its cognate receptor [46] and thus, can be assessed in a hemagglutination inhibition assay using chicken red blood cells in which a single dilution value indicates the minimum neutralization concentration [47]. Using the gH epitope-specific mAb 10C10 in this assay, agglutination was inhibited for all three chimeric influenza viruses at single dilution point and no inhibition was observed in wt virus (Figure 3A). Interestingly, the highest level of inhibition with 10C10 was observed against the HA/gH-13AA influenza virus. The results further demonstrate that gH peptide is exposed on the surface of the HA/gH chimeric viruses.

We next evaluated whether targeting the gH-peptide with the anti-gH antibody neutralizes infection of the chimeric influenza viruses. Neutralization assays were performed in triplicate using wt and chimeric influenza viruses pre-incubated with the anti-gH mAb 10C10, the PR8 HA site Sa-specific mAb PY102, murine HA stalk-specific mAb KB2 and human HA stalk-specific mAb CR9114 (Figure 3B). Both HA stalk-specific mAbs demonstrated similar neutralizing activity against wt and chimeric viruses indicating that the introduction of the gH sequence did not alter virus infection. Antibody 10C10 inhibited infection of the chimeric viruses expressing the gH peptide possibly by steric hindrance upon mAb binding. Notably, a similar trend observed in the hemagglutination inhibition study was found with HA/gH-13AA being more effectively neutralized by 10C10 compared to the chimeric viruses with the shorter epitope. Interestingly, an inverse trend was observed with mAb PY102, which showed decreased neutralizing activity as the length of the chimeric insert increased (wt HA > HA/gH-7AA > HA/gH-10AA > HA/gH-13AA). This may be due to shielding or disruption of the original Sa site of PR8 HA upon introduction of the gH peptides. In combination, these data indicated that the gH epitope was efficiently presented on the HA surface and could be potentially immunogenic.

### 3.3. Vaccination with Chimeric Influenza Viruses Elicits gH-Specific Antibodies

**Figure 4:**
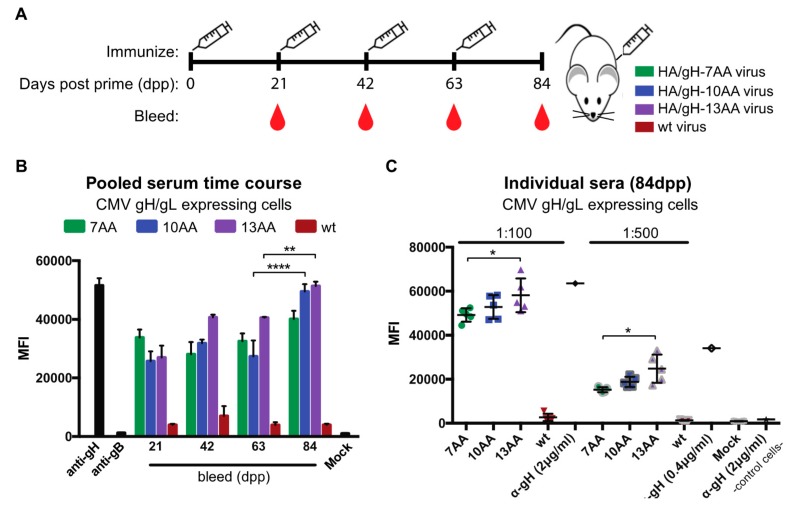
Chimeric influenza/CMV viruses generate a humoral response against CMV gH. (**A**) The immunization (syringe) and blood collection strategy for groups of five mice immunized with either chimera HA/gH viruses HA/gH-7AA (7AA), HA/gH-10AA (10AA), and HA/gH-13AA (13AA) or the wild type IAV virus (wt) lacking CMV components is depicted. Blood collection occurred prior to immunization. (**B**) Pooled sera from each immunization group was analyzed for specificity to cells expressing the CMV gH/gL dimer by measuring immunoglobulin binding followed by anti-mouse Ig-conjugated to Alexa647 (αmouse-Ig^Alexa647^) with flow cytometry. Controls included anti-gH (10C10) and anti-gB (2F4) (2 and 0.4 µg/mL) mAbs as well as cells probed with only αmouse-Ig^Alexa647^ (Mock). (**C**) Serum from individual mice at 84 days post prime (dpp) was subjected to flow cytometry analysis of CMV gH/gL-expressing cells. Anti-CMV gH mAb were utilized as a positive and negative control, respectively. Mock represents cells probed with αmouse-Ig^Alexa647^only and control cells refer to non-gH/gL expressing cells. The mean fluorescence intensity (MFI) was determined using FlowJo Software and the data points were from technical replicates with s.d. depicted. * *p* < 0.05, ** *p* < 0.01, **** *p* < 0.0001 (ANOVA).

In order to determine if the chimeric HA/gH influenza viruses generate an humoral antibody response to gH, the serum from mice immunized with HA/gH-7AA, HA/gH-10AA, HA/gH-13AA, or wt virus lacking the CMV gH peptide were evaluated for binding to CMV gH/gL expressing cells (Figure 4). The surface expression of the covalently-linked gH/gL complex modified with N-linked glycans allows for the analysis of sera specific binding to the native gH complex [48]. Groups of five mice were immunized several times with HA/gH-7AA, HA/gH-10AA, HA/gH-13AA, or wt virus and serum was collected at 21, 42, 63, and 84 days post prime (dpp) (Figure 4A). The pooled serum from individual animals was subjected to flow cytometry analysis utilizing U373 cells that stably express the gH/gL dimer [48] (Figure 4B). The sera from the control wt virus immunized mice and an anti-gB (CMV fusion protein) mAb revealed background levels of reactivity to the gH/gL cells (Figure 4B). Remarkably, serum antibodies against gH were generated following a single immunization for all chimeric viruses based on the increased antibody binding to gH/gL-expressing cells measured by mean fluorescent intensity (MFI). The results suggest that the chimeric viruses generated a humoral response that specifically recognizes the gH protein. Further, we observed an increase in serum antibody binding to gH/gL cells at later dpp bleeds of immunized animals. In fact, the pooled sera from day 84 of the HA/gH-10AA and HA/gH-13AA immunized animals yielded the highest fluorescent signal. The results demonstrate that antibodies specific for gH were generated using the HA/gH chimeric viruses, independent of peptide length as immunogens.

To examine the consistency of antibody responses to gH/gL cells of sera from animals with the highest binding values (Figure 4B), the individual serum samples from the 84dpp time point were tested by flow cytometry (Figure 4C). The serum from the wt control virus immunized animals revealed the background levels of serum binding, while the anti-gH 10C10 bound to the control cells and gH/gL-expressing cells acted as additional controls. Serum from HA/gH-7AA, HA/gH-10AA, and HA/gH-13AA virus immunized animals bound to gH/gL-expressing cells as observed by the significant increase in MFI compared to serum from mice vaccinated with wt control virus (Figure 4C). Additionally, the HA/gH-13AA sera demonstrated increased gH reactivity implying that the larger gH peptide induces an enhanced humoral response. Collectively, the data indicate that the introduction of a CMV gH peptide into the influenza HA molecule can elicit a reactive antibody response that recognizes native full-length gH protein.

### 3.4. Chimeric HA/gH Influenza Viruses Generate CMV Inhibitory Antibodies

**Figure 5:**
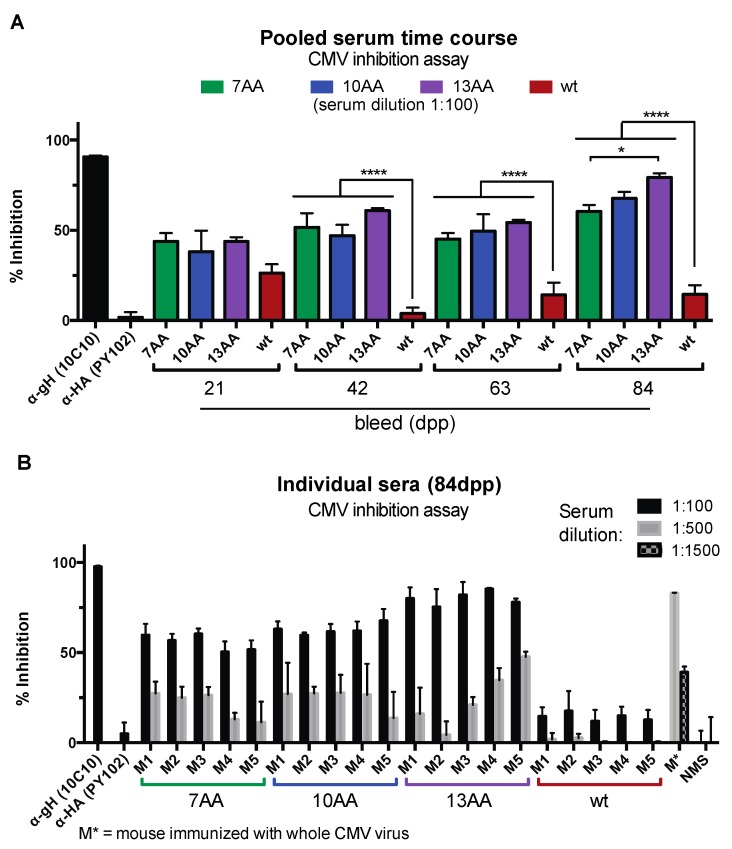
Chimera influenza/CMV viruses generate a CMV inhibition antibody response. (**A**) Pooled serum from each immunization group was analyzed in a CMV inhibition assay with the infection of AD169^BADrUL131^ (MOI: 0.5) pre-incubated with serum (1:100) in ARPE-19 cells. Cell infection was analyzed at 24 h post-infection using the Celigo fluorescence cytometer. Anti-CMV gH monoclonal antibody (mAb) (10C10) (2 µg/mL) and PR8 head specific mAb (PY102) (2 µg/mL) were utilized as positive and negative controls, respectively. (**B**) Serum from individual mice at 84 days post prime (dpp) was subjected to a CMV inhibition assay with infection of AD169^BADrUL131^ (MOI: 0.5) pre-incubated (1:100 and 1:500) with sera at specified dilutions in ARPE-19 cells. Pre-incubation with serum from a mouse immunized with the whole CMV (TB40/E) virus (M*) (1:500 and 1:1500), normal mouse serum (NMS) (1:100 and 1:500), and mAbs 10C10 and PY102 (2 µg/mL) were utilized as controls. The % inhibition was determined using NMS as 0% inhibition and no infected cells as 100% inhibition. Experiments were performed in technical replicates with the s.d. being depicted. * *p* < 0.05, **** *p* < 0.0001 (ANOVA).

Targeting viral envelope proteins gB and gH/gL complexes by monoclonal antibodies and polyclonal serum can effectively neutralize a CMV virus infection [49]. To test the inhibitory property of the serum elicited by vaccination with the chimeric influenza viruses, the sera from vaccinated animals were subjected to a high-throughput CMV infectivity assay using the AD169^BADrUL131^ reporter virus that encodes for yellow fluorescent protein (YFP) using similar conditions as previously characterized neutralization studies [25,33] (Figure 5). ARPE-19 epithelial cells were infected with AD169^BADrUL131^ pre-incubated with pooled serum from mice (1:100) immunized with HA/gH-7AA, HA/gH-10AA, and HA/gH-13AA, or wt control influenza virus lacking the gH peptide and analyzed 24 h post infection (hpi) for virus infection based on YFP positive cells using a Celigo cytometer (Figure 5A). Due to the non-specific inhibition of CMV observed with high concentrations of sera, the lowest dilution analyzed in the inhibition studies was 1:100. Virus infection was normalized based on normal mouse serum (NMS) at 0% inhibition and no infection at 100% inhibition. Given that the chimeric influenza viruses express a protein that includes a gH peptide from 7–13 aa, the humoral response would be highly specific against this peptide within the gH protein. Pre-incubation of AD169^BADrUL131^ with the anti-gH mAb 10C10 caused a significant inhibition of CMV infection as compared to the control anti-influenza HA mAb PY102. Importantly, the serum from all three chimeric influenza viruses significantly inhibited CMV infection at all time points (Figure 5A). The sera from the wt control influenza virus immunized animals slightly inhibited a CMV infection demonstrating the non-specific inhibitory activity of sera. Notably, vaccination with the HA/gH-13AA virus elicited the highest inhibition activity at 84dpp supporting the efficacy of the viral variant to generate a humoral immune response.

Next, we evaluated the sera from individual mice at 84dpp for their capacity to limit virus infection (Figure 5B). Serum pre-incubated with AD169^BADrUL131^ at different dilutions was analyzed for inhibition ability by measuring virus-infected cells at 24 hpi. The inhibition capacity of the sera was similar across the individual mice immunized with the respective chimeric viruses, with a range of 50%–80% inhibition at a 1:100 dilution (Figure 5B). Strikingly, the most effective serum to inhibit a CMV infection was from HA/gH-13AA virus immunized animals with ~80% reduction of CMV infection. On the other hand, the serum from the HA/gH-7AA virus immunized animals caused only a ~50–60% reduction in infection. These results support the model that the gH epitope in the HA/gH chimera can generate a humoral response with neutralizing capacity. The significant decrease in virus infection to ~80% is remarkable given that the anti-CMV antibodies are exclusively directed to a 7-13aa sequence within gH. In addition, it was impressive to observe that the 1:500 dilution of serum maintained 10%–50% inhibition. As a control, serum from a mouse immunized with the CMV strain TB40/E inhibited an AD169^BADrUL131^ infection of ARPE-19 cells by ~80% at a 1:500 dilution and ~40% at a 1:1500 dilution (Figure 5B). As expected, the putative polyclonal anti-CMV antibody response was more effective at inhibiting a CMV infection than serum with antibodies targeting a single region of gH. Collectively, the results demonstrate that a chimeric influenza virus containing an exogenous CMV peptide can generate a humoral response that can inhibit a CMV infection.

## 4. Discussion

CMV-associated diseases are a serious public health concern with currently no vaccine available to prevent disease. An effective vaccine would have a major impact in preventing CMV-related neurological disorders in newborns and significantly reduce CMV-associated morbidity and mortality in transplant recipients. Vaccines that elicit a targeted, protective immune response towards conserved epitopes could provide a means to prevent CMV infection ideally through the induction of humoral and cellular immunity against viral proteins [12,50]. An effective and targeted T cell response in combination with a strong humoral response against neutralizing epitopes would effectively limit virus proliferation and dissemination during primary and recurrent infections. There are numerous CMV vaccine strategies that target several proteins including the envelope protein gB, tegument proteins pp65, and immediate-early genes 1 and 2 to induce viral specific antibodies and T cells [11,12]. In addition, the V160 DISC candidate generated neutralizing antibodies and T cell immunity in several human primate models [51]. Yet, these vaccine candidates are designed based on the immune response generated from a natural CMV infection in humans. However, the immune response to a natural CMV infection does not completely protect against recurrent infections [52] and in fact, CMV continues to be transmitted from seropositive immune competent individuals and to the fetus of seropositive pregnant women [53]. We propose a CMV vaccine design that would target specific conserved viral domains that are underrepresented in seropositive individuals generating a protective immune response to protect against primary and recurrent infections. Chimeric influenza viral variants can be generated to induce a cellular immune response to CMV to provide complete protection against virus-associated diseases. Further, these chimeric influenza viruses can be evaluated efficacy against infection and dissemination in a congenital guinea pig cytomegalovirus infection model [54,55,56,57].

The epitope of the neutralizing anti-gH monoclonal antibody 10C10 is a highly conserved amino acid sequence (481-HTTERREIFIVET-493) among current strains of CMV and targeting this gH region would likely provide broad spectrum protection against CMV infection and proliferation [9,25]. Notably, this proof-of-principle study demonstrated that immunizing mice with influenza viruses expressing a single gH epitope elicited a humoral response that recognized gH/gL complexes in a binding assay and a neutralizing antibody response using established neutralization assays [25,30] (Figure 4 and Figure 5). The anti-gH antibody response is due to human CMV and not a possible concomitant murine CMV infection because there is only 12% identify between the two gH molecules and the specific gH peptide is not represented in murine CMV envelope proteins. Independent of the gH peptide length, all chimeric viruses induced a humoral response with enhanced CMV binding and neutralizing titers with the virus encoding the larger gH peptide (13AA) in the HA molecule (Figure 4 and Figure 5). This may be due because the longer peptide includes the entire anti-gH epitope, the gH peptide folded in a similar conformation as the native gH, and/or it is more exposed on the molecular surface. Strikingly, targeting a single peptide was capable of limiting a virus infection. These results are consistent with a previously engineered influenza viruses with peptides of HIV gp120 and *Plasmodium falciparum* cloned into the HA head generated a humoral response to the respective pathogens [58,59]. Further, variant influenza viruses containing exogenous peptides of other viruses including hepatitis C virus, respiratory syncytial virus, and HIV cloned into the neuraminidase, NS1, PB2, and M2 genes generated a humoral and adaptive immune response [29]. Thus, these chimeric viruses represent a platform to create additional chimeric viruses that generate both a humoral and cellular immune response to diverse CMV proteins.

The HA head domain is highly flexible and contains multiple immunogenic sites [41]. Importantly, potential insertion sites have been systematically mapped by a transposon mutagenesis approach [34,45]. In this study, we only utilized a single CMV epitope with 100% identity among all strains of CMV that was inserted into one antigenic site. It may be feasible to further expand on this approach by introducing additional CMV epitopes into other antigenic sites of diverse envelope proteins. In fact, additional peptides within gH and gB have been characterized to be targeted by neutralizing monoclonal antibodies [28,49]. The high similarity among the viral envelope proteins (e.g., gB~93%, gH~96%, UL130~98%, UL131a~99%) would allow for the humoral response to broadly prevent virus infection and dissemination [9,60,61]. The selection of a conserved CMV envelope protein would limit the escape of the antibodies raised to the specific sequence. In addition, a vaccine containing multiple conserved CMV epitopes could provide a highly protective immune response and prevent viral escape from the neutralizing humoral immune response.

A benefit of using influenza viruses as the vector for a CMV vaccination approach is that existing influenza vaccination platforms can be utilized, including inactivated split-virus vaccines, recombinant protein vaccines, as well as live-attenuated vaccination (LAIV) approaches [62]. LAIVs may be particularly interesting, since they elicit potent mucosal immune responses in children, which could be efficient at also blocking CMV infection [63]. Yet, the introduction of exogenous peptides into influenza variant may limit virus infection of host cells. To avoid this possibility, the sialic acid binding specificity of the variant would be maintained and rigorously evaluated in in vitro studies. An additional benefit, an influenza virus specific antibody response may be redirected towards conserved epitopes in the HA stalk domain, which is broadly cross-reactive and protective against a variety of influenza viruses [64]. However, some limitations of the influenza vaccine platform may be the length of peptide introduced into the HA protein, the chimeric HA molecule prevents the generation of the virus, or pre-existing immunity to influenza may limit the immune response to the exogenous peptide. In these cases, the peptide size and location within the HA molecule would be optimized in order to rescue an influenza variant. Alternatively, the peptide can be inserted into other influenza proteins (e.g., NA) that can generate a humoral response [29]. The influence of pre-existing immunity on an influenza-based vaccine candidate may be countered by utilizing a non-circulating influenza strain as the vaccine vector. Also, higher doses of the influenza vaccine may be required to circumvent pre-existing immunity to generate a robust immune response.

An important consideration for the use of this chimeric HA antigen in an LAIV context is the potential of reassortment with a wild-type strain and lose the potential to generate antibodies to the exogenous peptide. In this case, it would be necessary to study the reassortment potential of the virus before wide-spread use of such a vaccine. Yet, this concern would be alleviated by using inactivated virus vaccines, for which no antigenic changes or reassortment could occur and modifying the vaccination administration to a subcutaneous or intramuscular route. Alternatively, the peptide would be inserted into multiple sites within the HA molecule in which any reassorted viruses would be expected to maintain at least one exogenous peptide. Ideally, the influenza/CMV vaccine will only require one immunization. Our approach successfully utilizes an influenza virus vaccination platform to elicit a targeted humoral response towards CMV, and therefore supports future exploration into generating additional variant influenza viruses to ultimately provide an effective protective anti-CMV immune response.

## 5. Conclusions

Human cytomegalovirus (CMV) is an opportunistic virus that increases morbidity and mortality of individuals with an immunocompromised immune system. In this study, an influenza virus-based vaccine platform has been utilized to generate a CMV specific humoral response to a neutralizing epitope of the essential viral envelope protein gH. The specific humoral response to a single region of gH was able to inhibit a CMV infection in vitro supporting the utilization of the influenza virus vaccine platform. Collectively, the data has provided the proof of concept for exploration of influenza/CMV chimeras to generate an effective and protective anti-CMV immune response.
